# *Arabidopsis* replacement histone variant H3.3 occupies promoters of regulated genes

**DOI:** 10.1186/gb-2014-15-4-r62

**Published:** 2014-03-21

**Authors:** Huan Shu, Miyuki Nakamura, Alexey Siretskiy, Lorenzo Borghi, Izabel Moraes, Thomas Wildhaber, Wilhelm Gruissem, Lars Hennig

**Affiliations:** 1 Department of Biology and Zurich-Basel Plant Science Center, ETH Zurich, CH-8092 Zurich, Switzerland; 2 Department of Plant Biology, Uppsala BioCenter, Swedish University of Agricultural Sciences and Linnean Center for Plant Biology, SE-75007 Uppsala, Sweden; 3 Science for Life Laboratory, SE-75007 Uppsala, Sweden

## Abstract

**Background:**

Histone variants establish structural and functional diversity of chromatin by affecting nucleosome stability and histone-protein interactions. H3.3 is an H3 histone variant that is incorporated into chromatin outside of S-phase in various eukaryotes. In animals, H3.3 is associated with active transcription and possibly maintenance of transcriptional memory. Plant H3 variants, which evolved independently of their animal counterparts, are much less well understood.

**Results:**

We profile the H3.3 distribution in *Arabidopsis* at mono-nucleosomal resolution using native chromatin immunoprecipitation. This results in the precise mapping of H3.3-containing nucleosomes, which are not only enriched in gene bodies as previously reported, but also at a subset of promoter regions and downstream of the 3′ ends of active genes. While H3.3 presence within transcribed regions is strongly associated with transcriptional activity, H3.3 at promoters is often independent of transcription. In particular, promoters with GA motifs carry H3.3 regardless of the gene expression levels. H3.3 on promoters of inactive genes is associated with H3K27me3 at gene bodies. In addition, H3.3-enriched plant promoters often contain RNA Pol II considerably upstream of the transcriptional start site. H3.3 and RNA Pol II are found on active as well as on inactive promoters and are enriched at strongly regulated genes.

**Conclusions:**

In animals and plants, H3.3 organizes chromatin in transcribed regions and in promoters. The results suggest a function of H3.3 in transcriptional regulation and support a model that a single ancestral H3 evolved into H3 variants with similar sub-functionalization patterns in plants and animals.

## Background

Histones are abundant in most eukaryotic cells where they package DNA into chromatin. Dimers of histones H2A, H2B, H3 and H4 assemble into the histone octamer core to organize 147 bp of DNA into nucleosomes, the basic building blocks of chromatin. In recent years, much has been learned about how posttranslational modifications of histones affect chromatin, such as modifying inter-nucleosomal contacts or nucleosome stability. It is now also well established that incorporation of histone variants can result in formation of chromatin with particular properties [1-3]. In centromeric chromatin, for example, canonical H3 is replaced by the CenH3 variant forming tetrameric hemisomes instead of the conventional octameric nucleosomes [4]. Another H3 variant that was found in many eukaryotes is the histone replacement variant H3.3 [1]. In contrast to the canonical histone H3.1, incorporation of metazoan histone H3.3 into chromatin is mostly replication-independent [5]. The sequences of metazoan H3.1 and H3.3 differ only at position 31 in the amino-terminal tail and at positions 87 to 90 in the core histone fold [6]. Genome-wide profiling of H3.3 in *Drosophila* and mammalian cells revealed specific incorporation into the gene body of active genes, into promoter regions of both active and inactive genes, as well as into regulatory elements [7-10], supporting the idea that H3.3 has a role in transcription [11]. Unexpectedly, recent data have revealed H3.3 enrichment also at silent loci in pericentric heterochromatin and in telomeres [9,12], and have shown a requirement of H3.3 for correct heterochromatin formation in mouse embryos [13]. H3.3 is incorporated into pericentric heterochromatin during S phase when pericentric repeats are transcribed; therefore, it was suggested to have a role in the initial formation of double stranded RNA-dependent heterochromatin [13]. Recently, mutations in an H3 replacement pathway were connected to pathogenesis of glioblastoma multiforme, a lethal brain tumor [14].

Replication-coupled and replication-independent (replacement) H3 histone variants evolved independently in animals, plants, basidiomycetes, and alveolates [15]. Similar to the replacement H3 variant H3.3 in animals, *Arabidopsis* H3.3 differs from H3.1 at positions 31, 87 and 90 but also at some additional positions [16]. There are three H3.3 genes in *Arabidopsis* (*At4g40030*, *At4g40040*, and *At5g10980*) [16,17], which are expressed constitutively in a replication-independent manner [16,18]. However, it is currently unknown if *Arabidopsis* H3.3 has the same epigenomic properties as the animal H3.3 variant. Recently, the genome-wide distribution of the *Arabidopsis* H3.3 protein At4g40040 was reported and, similar to animal H3.3, was preferentially found in the bodies of transcribed genes [19,20]. In contrast to animal H3.3, however, plant H3.3 was not generally detected outside of gene bodies. Therefore, we decided to re-analyze H3.3 profiles using a protocol with increased sensitivity and mono-nucleosomal resolution. This protocol revealed H3.3 enrichment not only in gene bodies as previously reported but also at a subset of promoter regions and downstream of the 3′ ends of active genes. In particular, promoters containing GA motifs were targeted for H3.3 incorporation regardless of their activity. Our data suggest that the evolutionary constraints behind the evolution of animal and plant H3 histone variants are more general than previously assumed and may contribute to transcriptional regulation.

## Results

### Histone H3.3 is targeted to euchromatin

**Figure 1:**
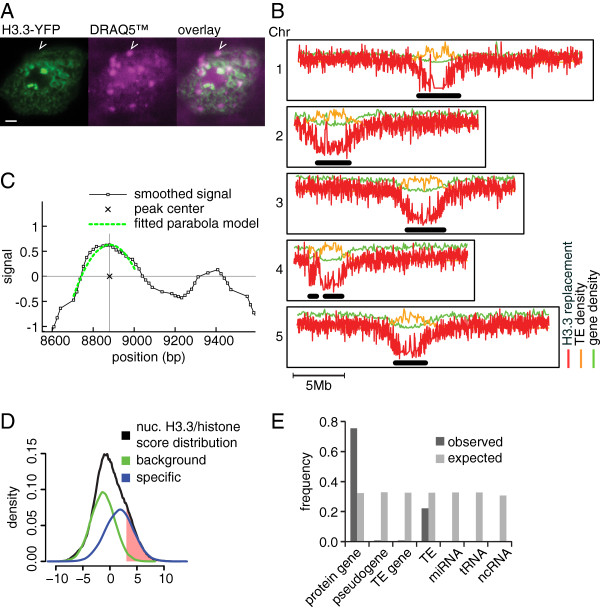
**Genome-wide profiling of H3.3 in *Arabidopsis*. (A)** H3.3-YFP is targeted to the nucleus. Confocal images of nuclei from *Arabidopsis* roots showing H3.3-YFP (green) and a DNA-counterstain with DRAQ5 (red). Open arrowheads mark heterochromatic chromocenters. Scale bars: 2 μm. **(B)** Genome-wide profiles of H3.3 enrichment (red), transposable element (TE; yellow) and gene density (green). Bars mark centromeric and pericentric heterochromatin. **(C)** Nucleosome positions were detected by fitting the smoothed ChIP-chip signal (black line with squares) to a parabola model (green dashed line). The position of the maximum of each fitted parabola peak was regarded as the center of a nucleosome (cross). **(D)** Deconvolution of H3.3 enrichment at positions of nucleosomes (black) into a background (green) and a specific (blue) component using a mixture model. The red area indicates nucleosomes that have higher than 75% probability belonging to the specific component. **(E)** Comparison of observed and expected distribution of H3.3 nucleosomes across different genomic features (that is, protein coding gene, pseudogene, transposable element gene, transposable element, microRNA (miRNA), tRNA, non-coding RNA (ncRNA)).

To map histone H3.3 distribution in the *Arabidopsis* genome, we generated *Arabidopsis* lines expressing H3.3 tagged by yellow fluorescence protein (YFP; H3.3-YFP). Among several transformed lines that were phenotypically indistinguishable from wild-type control plants (Figure S1 in Additional file 1) a single line was selected for detailed analysis. Note that total H3.3 expression was not increased in this line (Figure S1 in Additional file 1). The distribution of H3.3 signals was first investigated using confocal laser scanning microscopy. At the cytological level, YFP signals showed speckle-like patterns throughout the nucleoplasm but were weaker in the heterochromatic chromocenters (Figure 1A, open arrowhead), similar to previous observations [21]. To investigate H3.3 distribution at a genomic level, we generated a H3.3 enrichment map at single nucleosome resolution by native chrommatin immunoprecipitation (ChIP)-chip using input chromatin digested by micrococcal nuclease (MNase) to mono-nucleosomal fragments. DNA from immunoprecipitated chromatin was amplified and hybridized onto AGRONOMICS1 Affymetrix whole genome tiling arrays (see Materials and methods for more details). An H3.3 enrichment score of each probe position was calculated as H3.3 density normalized to nucleosome density derived from a control MAB3422 anti-histone ChIP (see Materials and methods). The resulting map showed that H3.3 was abundant on gene-rich euchromatic arms and was absent from the gene-poor heterochromatic pericentric regions (Figure 1B), consistent with the cytological observation and previous reports [19,20]. To verify our ChIP-chip results, we performed independent ChIP-quantitative PCR (qPCR) experiments for selected loci. ChIP-chip and ChIP-qPCR measurements were in good agreement with each other (Figure S2 in Additional file 1).

To investigate H3.3 distribution at the nucleosome level, we located well-positioned single nucleosomes detected by either H3.3 or histone control ChIP by fitting the ChIP-chip signals to a parabola model (Figure 1C). Total detectable nucleosomes were combined from the two lists, resulting in 138,609 positioned single nucleosomes. To determine a H3.3 enrichment score for each nucleosome, we calculated the median of H3.3 ChIP-chip scores normalized to the control histone ChIP-chip scores in a window of 147 bp around the detected nucleosome centers. Similarly, we calculated the background noise level of each nucleosome using the median of a control IgG ChIP-chip score normalized to the histone ChIP-chip score. We then used two criteria to select H3.3 nucleosomes. First, the nucleosome H3.3 enrichment score had a higher than 75% probability of belonging to the specific component in a two-component mixture model (Figure 1D). Second, the nucleosome H3.3 enrichment score was higher than two times the background noise score. Under these high stringency criteria, we identified 28,220 H3.3 nucleosomes in the genome (Table S1 in Additional file 2), of which the majority (>99.6%) were located on the euchromatic chromosome arms and in close proximity to annotated genes. We associated H3.3 nucleosomes with the nearest genomic feature (that is, protein coding gene, pseudogene, transposable element gene, transposable element, microRNA, tRNA, non-coding RNA) if the distance did not exceed 2,000 bp. Using this criterion, 26,216 (92.9%) H3.3 nucleosomes were associated with genomic features. Out of all 20,381 genomic features that had closely associated H3.3 nucleosomes, 15,378 (75.5%) were protein coding genes, which is a 2.3-fold enrichment over random sampling (*P*-value = 2.21 × 10^-11^; two-tailed *t*-test; Figure 1E). All other genomic features were only rarely associated with H3.3 nucleosomes (Figure 1E).

### H3.3 is enriched at promoters and around transcriptional termination sites in a transcription-dependent manner

**Figure 2:**
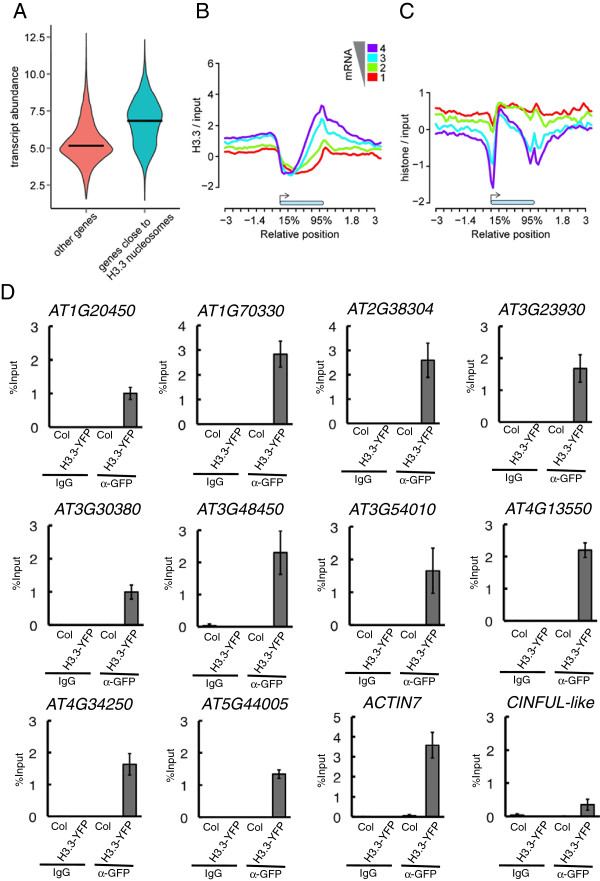
**H3.3 is enriched at transcribed genes and has a strong 3′ bias. (A)** Genes with at least one H3.3 nucleosome (right) have higher transcript abundance than genes without H3.3 (left). Horizontal bars indicate median values. Expression data are from [22] and in logarithmic scale. **(B,C)** H3.3 nucleosome (B) and total nucleosome (C) occupancy were measured as H3.3-YFP-ChIP and histone-ChIP signals, respectively. Metagene plots across gene bodies (blue bar) were constructed between -3 kb and +3 kb. Genes were grouped according to transcript abundance from low (red) to high (violet). **(D)** Validation of H3.3 at promoters by ChIP-qPCR. 5′ Upstream regions of 10 genes that had H3.3-enrichment at promoters in the ChIP-chip experiment were tested. *ACTIN7* and a *CINFUL*-like locus served as controls. Error bars represent standard error of mean, n = 3. GFP, green fluorescent protein.

Considering the significant H3.3 nucleosome enrichment at protein-coding genes, we asked whether H3.3 enrichment was associated with transcription. To avoid ambiguity, we restricted the analysis to genes with only a single annotated splice variant. Firstly, for the genes with at least one closely associated H3.3 nucleosome, the transcript abundance was significantly higher than for those genes without a H3.3 nucleosome in close proximity (Figure 2A; *P*-value <2.2 × 10^-16^, one-tail Wilcoxon test). We next grouped all the genes into four bins according to their relative transcript abundance, and for each group averaged H3.3 profiles were plotted along genes (Figure 2B; Figure S3 in Additional file 1). This revealed a positive correlation between H3.3 and transcript abundance, especially at promoters and transcription termination sites (TTSs). Genes with no detectable transcripts (bin 1) showed uniform depletion of H3.3 from gene bodies (Figure 2B, red line; Figure S3 in Additional file 1), while genes with the highest transcript abundance (bin 4) had the highest H3.3 enrichment at both promoter and TTSs (Figure 2B, purple line; Figure S3 in Additional file 1). Notably, H3.3 enrichment extended considerably downstream of the TTS of highly transcribed genes. Furthermore, H3.3 enrichment around TTSs changed more strongly with transcript abundance than H3.3 enrichment at promoters. Relative to TTSs, the 5′ proximal regions of protein-coding genes were generally depleted of H3.3 independent of their transcription activity. H3.3 and histone density were generally inversely correlated (Figure 2C) with highest histone density at inactive genes (Figure 2C, red line) and in the 5′ coding region of active genes (Figure 2C, purple line). H3.3-enriched regions consisted in most cases at both promoters and TTSs of only one identified H3.3 nucleosome. This high-confidence H3.3 nucleosome was usually flanked by regions with substantial but not significant H3.3 incorporation. Because the presence of H3.3 at promoters was not previously observed in plants, we validated this ChIP-chip result by independent ChIP-PCR experiments using 10 genes with promoter H3.3. Indeed, a robust signal of H3.3 was detected for all tested promoters (Figure 2D; Figure S4 in Additional file 1).

Together, these results show that H3.3 deposition targets mainly promoters and the region around the TTS, and that the H3.3 levels in both regions positively correlate with transcriptional activity of genes.

### H3.3 in promoters co-localizes with RNA Polymerase II

**Figure 3:**
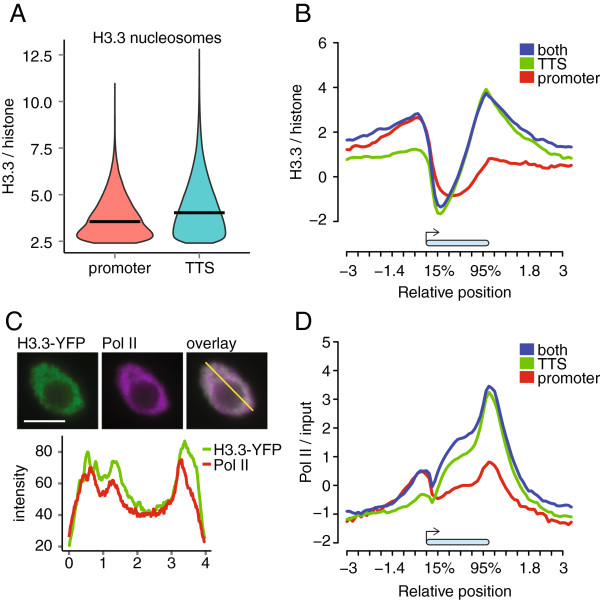
**H3.3 presence coincides with RNA Polymerase II distribution. (A)** H3.3 enrichment scores of promoter-associated and TTS-associated H3.3-enriched nucleosomes. Horizontal bars indicate median values. **(B)** Metagene plots across gene bodies (blue bar) were constructed between -3 kb and +3 kb for H3.3 signals normalized to histone signals. Here and in subsequent metagene plots, positions plotted on the x-axis are relative to annotated transcription start site and TTSs and given in kilobases for upstream and downstream sequences and as percentages for gene bodies. Genes were grouped according to the presence of H3.3-enriched nucleosomes in the promoter (red), close to the TTS (green) or both (blue). **(C)** Immunocytological detection (upper) of H3.3 (left) and RNA Polymerase II (Pol II; center). An overlay is shown at the right. Scale bars: 5 μm. The diagram (lower) shows H3.3 (green) and Pol II (red) signals along a section through the nucleus (yellow line in the overlay image). **(D)** Metagene plot of Pol II across gene bodies (blue bar) between -3 kb and +3 kb. Pol II ChIP-chip data are from [23]. Genes were grouped according to the presence of H3.3-enriched nucleosomes in the promoter (red), close to the TTS (green) or both (blue).

Because we found H3.3 in promoters and around TTSs, we asked whether all genes have equal H3.3 levels at both locations. We classified each H3.3 nucleosome as either promoter- or TTS-associated. Among the H3.3 nucleosomes associated with genes, 4,293 and 17,897 were unequivocally promoter- and TTS-associated, respectively (Table S1 in Additional file 2). Promoter-associated H3.3 nucleosomes were significantly less enriched than TTS-associated H3.3 nucleosomes (Figure 3A; *P*-value <2.2 × 10^-16^, one-tail Wilcoxon test). Therefore, H3.3 incorporation occurs with the highest frequency at the TTSs of genes. In total, we identified 1,891 genes with only promoter-associated H3.3 nucleosomes, 10,447 genes with only TTS-associated H3.3 nucleosomes, and 3,012 genes with both promoter- and TTS-associated H3.3 nucleosomes (Figure 3B; Figure S5 in Additional file 1). Specifically, enrichment of promoter-associated H3.3 nucleosomes was highest 300 bp upstream of the transcription start site (TSS), while the enrichment of TTS-associated H3.3 nucleosomes had a maximum directly at the TTS of the gene.

In *Drosophila*, H3.3-containg nucleosomes repackage DNA following the passage of elongating RNA Polymerase II (Pol II) during the transcription of genes [24], but H3.3-containg nucleosomes were also reported in promoters [10]. We hypothesized that in *Arabidopsis* the role of H3.3 in nucleosomes would also be coupled to Pol II activity. Indeed, we found that in *Arabidopsis* H3.3 co-localizes with Pol II in the nucleus (Figure 3C). At the gene level, we expected that only TTS-associated but not promoter-associated H3.3 would reflect Pol II presence. However, we found that H3.3 at promoters as well as around TTSs was associated with substantial Pol II binding (Figure 3D; Pol II data were from [23]), in particular on the subset of genes with H3.3 nucleosomes in their promoters. No considerable Pol II binding to promoter regions was observed for genes without H3.3 nucleosomes upstream of the TSS (Figure 3D). Although Pol II binding outside of transcribed regions is not well documented, a high-resolution study in *Saccharomyces cerevisiae* had reported a Pol II peak at -100 bp of the TSS for moderately expressed genes [25]. It should be noted that in *Arabidopsis*, Pol II occupancy at promoters was much lower than occupancy around TTSs. In contrast, H3.3 levels differed much less between these two positions (Figure 3B,D). These observations support the notion that H3.3 nucleosomes, both at promoters and around TTSs, are associated with the presence of Pol II in *Arabidopsis*.

### H3.3 incorporation at promoters reflects strong transcriptional regulation

**Figure 4:**
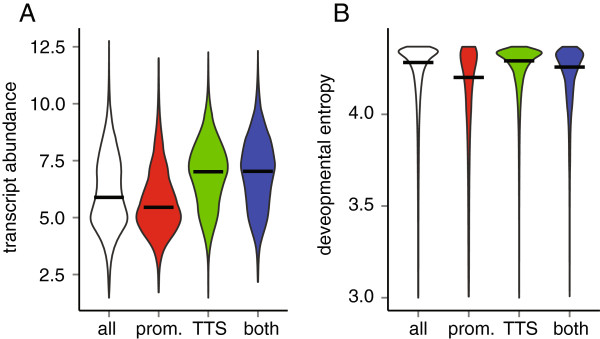
**Genes with H3.3-enriched nucleosomes in the promoter have weak expression but strong regulation. (A)** Transcript abundance for all genes (white), genes with H3.3-enriched nucleosomes in the promoter (red), close to the TTS (green) or both (blue). Expression data are from [22] and in logarithmic scale. **(B)** Expression entropy for all genes (white), genes with H3.3-enriched nucleosomes in the promoter (red), close to the TTS (green) or both TTS (blue). Expression entropy is based on data from [26]. Horizontal bars indicate median values.

We next asked whether H3.3 enrichment at promoters and around TTSs was similarly related to transcription. Genes with H3.3 enriched only around TTSs and genes with H3.3 enriched at both promoters and around TTSs had significantly higher transcript abundance than the genome-wide median (Figure 4A; *P*-value <2.2 × 10^-16^, one-tail Wilcoxon test), consistent with the general trend shown in Figure 2A. Interestingly, however, genes with H3.3 only at promoters were not particularly strongly expressed and had even lower transcript levels than the genome-wide median (Figure 4A; *P*-value <2.2 × 10^-16^, one-tail Wilcoxon test). Therefore, H3.3 presence around TTSs is a better predictor for high transcript abundance than H3.3 presence at promoters.

In budding yeast, Pol II was found upstream of inactive genes that could be rapidly activated upon exit from the stationary phase [27]. We asked whether the *Arabidopsis* genes with H3.3-enriched nucleosomes and Pol II at their promoters are strongly regulated. To test this hypothesis, we calculated for each gene an expression entropy using collections of *Arabidopsis* transcript profiling data [26,28] (Figure 4B; Figure S6 in Additional file 1). Expression entropy is a measure for the extent of transcriptional regulation, with small values indicating a high extent of regulation. Consistent with our hypothesis, genes with H3.3 enriched at their promoters had significantly smaller expression entropies than the genome-wide median (*P*-value = 8.89 × 10^-5^, one-tail Wilcoxon test). In contrast, genes with H3.3 enriched only around the TTS had significantly larger expression entropies than the genome-wide median (*P*-value <2.2e-16, one-tail Wilcoxon test). Genes with H3.3 enriched at both promoters and around TTSs had reduced expression entropies, which were, however, much larger than those of genes with H3.3 enriched only at their promoters. These results support our hypothesis that genes with H3.3 enriched at their promoters are subject to strong transcriptional regulation.

**Figure 5:**
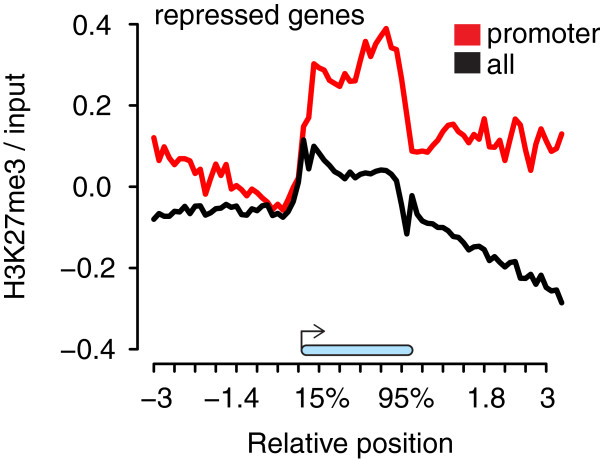
**Genes with H3.3-enriched nucleosomes in the promoter are marked by H3K27me3.** Metagene plot of H3K27me3 for all repressed genes (black) and for repressed genes with promoter H3.3 (red) across gene bodies (blue bar) between -3 kb and +3 kb. H3K27me3 ChIP-chip data are from [29].

Reduced expression entropy and strong developmental regulation are characteristic for Polycomb group protein target genes enriched with H3K27me3 [29]. Therefore, we tested whether promoter H3.3 was associated with increased H3K27me3. Indeed, genes with promoter H3.3 had considerably higher H3K27me3 over gene bodies than other inactive genes (Figure 5). This is consistent with findings in mammalian embryonic stem cells, where HirA-dependent H3.3 deposition was found to facilitate PRC2 recruitment [30], and in plants, where Asymmetric Leaf1 was found to interact with both HirA and PRC2 [31,32]. However, H3K27me3 was much lower on genes with promoter H3.3 than on well-established PRC2 targets (Figure S7 in Additional file 1) and established PRC2 targets were not enriched in the set of genes with promoter H3.3 (39.6% versus 37.7% on all inactive genes). Thus, promoter H3.3 is associated with H3K27me3 in plants but it remains to be investigated whether it also has a role in Polycomb group protein function in leaves, for example, by directly facilitating PRC2 recruitment in particular plant cell types similar to the situation in mammalian embryonic stem cells.

### H3.3 at promoters is independent of H2A.Z

In mammalian cells, DNA of active promoters is often bound by nucleosomes containing both H3.3 and H2A.Z, and it was suggested that combined incorporation of both histone variants could affect the access of transcription factors [10,33]. We asked whether H3.3-enriched nucleosomes at promoters of *Arabidopsis* genes also coincided with H2A.Z. Contrary to the finding in mammalian cells, plant H2A.Z [34] is mostly enriched downstream of the TSS (Figure S8 in Additional file 1, black line), where H3.3 levels are low (Figure S8 in Additional file 1, red line). This non-overlapping localization of H3.3 and H2A.Z on different sides of the TSS is consistent with earlier observations of H3.3 depletion at sites enriched with H2A.Z [19,20] and suggests that H3.3-H2A.Z-containing nucleosomes are not highly abundant at *Arabidopsis* gene promoters.

### H3.3 at promoters negatively correlates with DNA methylation

**Figure 6:**
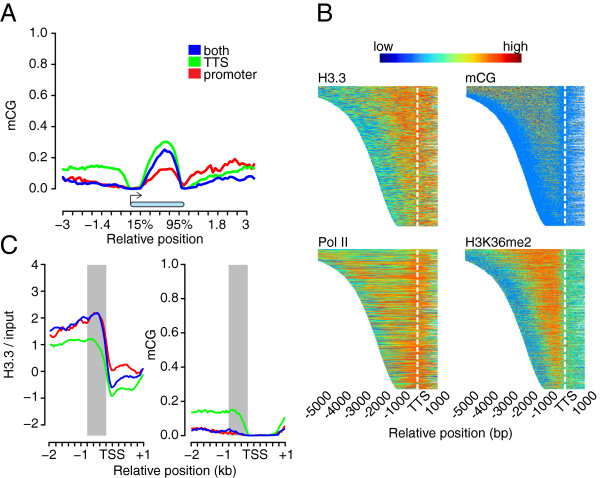
**DNA mCG methylation at promoters does not coincide with H3.3 enrichment. (A)** Metagene plot of mCG across gene bodies (blue bar) between -3 kb and +3 kb. mCG data are from [40]. Genes were grouped according to the presence of H3.3-containing nucleosomes in the promoter (red), close to the TTS (green) or both (blue). **(B)** H3.3, mCG, Pol II and H3K36me2 profiles around TTS. Signals were plotted from the TSS up to 1 kb proximal to the TTS for 5,000 random sampled genes ordered by their gene body lengths. For genes that are longer than 5 kb, only 5 kb of the gene body are shown. Epigenome profiles are represented by a heat map color code with red and blue representing highest and lowest values, respectively. White dashed lines indicate the TTS positions. Data are from [23,29,40]. **(C)** Metagene plot of H3.3 signal (left) and mCG (right) between -2 kb and +1 kb of the TSS. mCG data are from [40]. Genes were grouped according to the presence of H3.3-containing nucleosomes at the promoter (red), close to the TTS (green) or both (blue). Grey areas indicate -800 to -200 bp of the TSS.

In mammalian cells, DNA methylation was shown to either facilitate or exclude H3.3 loading in different genomic contexts [35,36]. *Arabidopsis* H3.3 is largely excluded from pericentric heterochromatin (Figure 1B) where cytosine methylation in CpG contexts (mCG) is maximal [37]. However, similar to H3.3, mCG is also found in bodies of expressed *Arabidopsis* genes [37]. It was observed that *Arabidopsis* H3.3 preferentially associates with mCG [19]. Indeed, genes with H3.3 enriched around the TTS had higher gene-body mCG levels than genes without H3.3 enrichment (Figure 6A), possibly reflecting their higher transcription rates. However, while mCG had a very good spatial overlap with H3K36me2 (Figure 6B), a histone modification known to associate with transcription elongation [29], H3.3 enrichment around TTSs had a stronger 3′ bias in the gene body than either gene-body mCG or H3K36me2 (Figure 6B). Moreover, while gene-body mCG and H3K36me2 do not extend beyond the TTS, H3.3 peaked at the TTS and extended considerably beyond, reflecting the Pol II signal extending beyond TTS (Figure 6B). Note that Pol II has also been found associated with DNA several hundred base pairs beyond the poly(A) site on many human genes [38,39]. Therefore, incorporation of H3.3 into nucleosomes at TTSs does not seem to be directly associated with gene-body DNA methylation; the mCG distribution reflects mostly the H3K36me2 and elongating Pol II distribution, while H3.3 enrichment may reflect terminating Pol II complexes (Figure 6B).

Furthermore, genes with H3.3 enriched at promoters lack almost any mCG, especially in the -800 to -200 bp window where H3.3 is highest (Figure 6C). In contrast, mCG levels are elevated in the same window for promoters without H3.3 enrichment (Figure 6C). The difference in mCG levels in this window between the two groups of promoters is highly significant (genes with only promoter H3.3-enriched nucleosomes compared to genes with only TTS H3.3 nucleosomes, *P*-value = 1.46 × 10^-10^, Wilcoxon’s rank test, one tail). Therefore, H3.3 and mCG appear to exclude each other at promoters.

In summary, H3.3 enrichment in nucleosomes does not strongly correlate with mCG in gene bodies and is negatively correlated with mCG at promoters.

### GA promoters are targeted by H3.3

**Figure 7:**
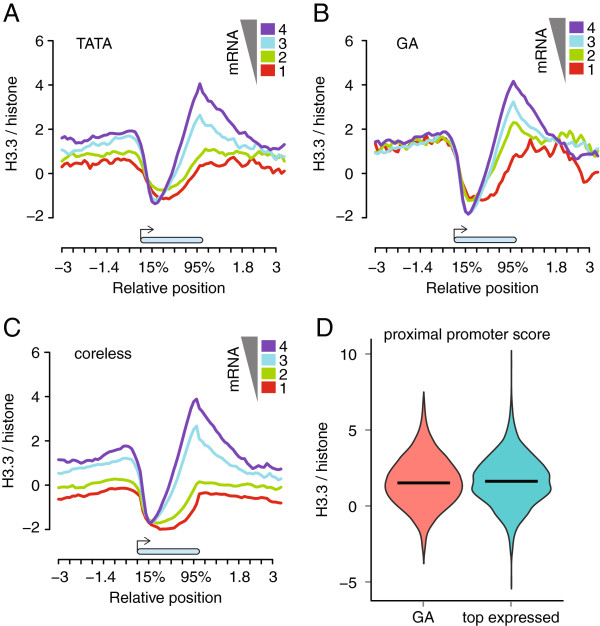
**Promoter *cis*-regulatory sequences affect local H3.3 incorporation. (A-C)** Metagene plots of H3.3 signals across gene bodies (blue bar) between -3 kb and +3 kb for genes with promoters that carry TATA elements **(A)**, GA elements **(B)** or neither TATA nor GA elements (coreless) **(C)**. Genes were first grouped according to transcript abundance, and genes with promoters of each type are presented separately. **(B)** H3.3 signals at promoters with GA elements (red) or promoters of the top 25% most strongly expressed genes (blue).

*Arabidopsis* promoters can be categorized into three major groups depending on the core promoter element, that is, TATA, GA and coreless [41]. Following this classification, we selected three non-overlapping groups of genes (TATA, 3,471; GA, 2,456; coreless 16,270) and asked whether different promoters are differentially targeted by H3.3. Similar to what was observed for all genes (Figure 2B), H3.3 enrichment at TATA and coreless promoters increased with increasing transcript abundance of genes (Figure 7A). Strikingly, however, for GA promoters such a correlation was absent: regardless of the transcript abundance, all GA promoters had similar H3.3 signals (Figure 7A), comparable to that of promoters of highly expressed genes in the genome (bin 4; purple line in Figure 2B). In fact, the H3.3 enrichment at -800 to -200 bp of annotated TSSs was not statistically different between GA promoters and promoters of the 25% most strongly expressed genes (Figure 7B; *P*-value = 0.087, Wilcoxon’s rank test, two-tail). These results show that GA promoters are special because they are targeted by H3.3 incorporation regardless of transcriptional activity.

### H3.3 nucleosomal DNA is more accessible

**Figure 8:**
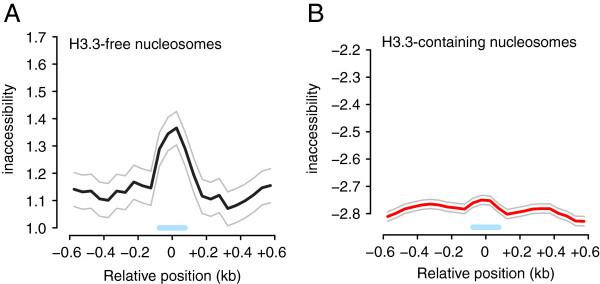
**DNA at H3.3-enriched nucleosomes is highly accessible. (A,B)** Metagene plots of inaccessibility of DNA around nucleosomes without (A) or with (B) H3.3 between -600 bp and +600 bp. Blue bars, nucleosome covered region. Data are from [22]. Shown are mean (black and red) and 95% confidence intervals (grey).

Mammalian H3.3 nucleosomes greatly impair higher-ordered chromatin folding [33], suggesting that *in vivo* DNA bound by H3.3 nucleosomes might be more accessible than DNA bound by H3.1 nucleosomes. To test this hypothesis, we compared the accessibility of DNA [22] around H3.3-free and H3.3-containing nucleosomes. Because H3.3 nucleosomes are highly enriched on euchromatic chromosome arms, we restricted our analysis of H3.3-free nucleosomes to those in euchromatin. H3.3-free nucleosomes were in generally less accessible environments than H3.3-containing nucleosomes (Figure 8A,B). Furthermore, H3.3-free nucleosomes protected the nucleosomal DNA as evident from the local increased inaccessibility relative to the flanking sequences (Figure 8A). Although H3.3-containing nucleosomes protect the DNA to some extent (Figure 8B), this effect is much smaller than that for the H3.3-free nucleosomes (Figure 8A). Therefore, DNA bound by H3.3-containing nucleosomes is both generally and locally more accessible.

## Discussion

H3.3 is a histone variant that differs only in four or five amino acids from the canonical H3.1 but it can have profound effects on chromatin functionality. Earlier studies had suggested that animal H3.3-containing nucleosomes isolated from native chromatin are less stable *in vitro*[42] but more recent reports indicate that H3.3 *per se* does not affect stability of mononucleosomes [33]. Instead, animal H3.3 appears to mainly impair higher-order chromatin folding. Our finding that DNA flanking H3.3-containing nucleosomes in plant chromatin is much more accessible to DNase I than DNA flanking H3.3-free nucleosomes is consistent with the notion that H3.3 interferes with higher-order chromatin folding. In addition, H3.3 deposition, which disrupts chromatin, could directly result in increased accessibility of DNA at H3.3-enriched nucleosomes.

H3.3 incorporation is generally thought to be associated with the transcription initiation and/or elongation activities of Pol II in animals and is highest in gene bodies [7,9,11,43-46]. We have used high-resolution mapping of the *Arabidopsis* replacement histone variant H3.3 and found that in plants H3.3 is located in gene bodies as well and shows a positive correlation with transcriptional activity, which is consistent with earlier reports [19,20]. In contrast to the general consent on a positive correlation between transcriptional activity and H3.3 levels in animals, the actual distribution of H3.3 over the gene body remains controversial. Reported patterns of H3.3 distribution range from 5′-biased in *Drosophila*[7,8] to 3′-biased in mammals [9,10]. Although the choice of methods may have contributed to the reported H3.3 patterns in animals, the observed patterns could also reflect different nucleosome turnover rates during transcription elongation in different organisms or different cellular environments [7]. It is noteworthy that Pol II can transcribe through hexasomal nucleosomes *in vitro* after eviction of a single H2A/H2B dimer while the H3/H4 tetramer remains associated with the DNA [47,48]. Complete dissociation of histone octamers from the DNA appears to be restricted to highly transcribed genes with multiple elongating Pol II molecules. Thus, transcription *per se* might not be sufficient to cause H3 replacement. This is consistent with the non-uniform and specific H3.3 patterns along gene bodies. Our results in *Arabidopsis* revealed a strong 3′ bias along gene bodies when examining plant H3.3 patterns by normalizing either to input or histone density, similar to earlier observations [19,20]. Our data also show high histone density at the 5′ end and a sharp decrease towards the 3′ end in the gene bodies, demonstrating that low H3.3 levels at 5′ ends were not caused by local loss of nucleosomes during the chromatin preparation. Thus, H3 exchange in plants and animals appears not to be linked to Pol II passage *per se* but appears to be restricted to specific phases of the transcription process.

In animals, H3.3 levels correlate with transcriptionally active, Ser-5 phosphorylated Pol II, transcription initiation site-related mono- and tri-methylation of histone 3 lysine 4 (H3K4me1, H3K4me3), and transcription elongation-related tri-methylation of histone 3 lysine 36 (H3K36me3) [9]. In *Arabidopsis*, H3K4me3 is found proximal to TSSs [29] where H3.3 is mostly absent, suggesting that the functional relevance between H3.3 localization and Pol II transcription initiation is different between plants and animals. On the other hand, *Arabidopsis* H3.3 is highly enriched for H3K36me2 [49], the histone mark thought to be associated with transcription elongation in *Arabidopsis*[29]. This suggests a connection between H3.3 and elongating Pol II similar to the situation in animals. In addition, our finding in *Arabidopsis* that H3.3 localization extends considerably beyond the TTS indicates an even stronger connection between H3.3 and Pol II transcription termination.

In addition to gene bodies, we found H3.3 also in plant promoters. H3 replacement at active promoters has been reported for mammals [10], and it is frequent in yeast, where H3 replacement is found more often in promoters than in gene bodies [50-52]. Although yeast does not have separate H3.1 and H3.3 genes, it does have mechanisms for replication-independent H3 replacement [53]. However, H3 replacement at promoters in yeast is not strongly correlated with transcription initiation or Pol II promoter occupation [50-52]. In *Arabidopsis*, we found H3.3 at promoters of both active and inactive genes. It is possible that H3.3 is incorporated at promoters independently of transcription or that it is a footprint of past transcription activity of the gene. Since we could also find Pol II associated with H3.3-enriched promoters, it is possible that RNA Pol II promoter occupation or transcription caused local H3.3 incorporation. Indeed, transcription of promoter-associated short RNAs is more ubiquitous than initially thought during transcription activation [54,55]. H3.3 insertion at promoters could also be a consequence of abortive rounds of transcription initiation that occur at repressed promoters [44] and can in turn poise the genes for transcription activation upon future induction [9]. Alternatively, H3.3 might be targeted to promoters by a transcription-independent mechanism as proposed for yeast [50-52] to facilitate binding of inactive Pol II to promoters of strongly regulated genes, such as genes that are activated upon exit of yeast from stationary phase [27]. The increased accessibility of DNA at H3.3-containing nucleosomes, likely reflecting reduced higher-order chromatin folding [33], suggests that the enrichment of H3.3 at promoters could allow easier access of transcription factors or the Pol II transcription initiation complex to the DNA template. Indeed, H3.3 incorporation can promote gene activation [46,56] or prime genes for subsequent activation [33]. Our data revealed that GA motif-containing promoters are targeted by H3.3 even when repressed and that this preferential targeting coincided with higher expression dynamics of these genes. These observations implicate H3.3 in potentiating transcription activation in plants similar to the binding of inactive Pol II to promoters of regulated genes in yeast.

## Conclusions

Animal and plant H3 variants evolved independently [15,57], but H3.3 incorporation patterns in plants and animals and replication-independent H3 deposition in yeast [51] have many similarities. Replication-independent chromatin assembly is essential for life, but separate H3.1 and H3.3 variants appeared independently in animals and plants. The evolutionary history of histone genes is still a matter of debate [15], but it is likely that the ability to affect higher-order chromatin structure by incorporation of specific histone variants confers major selective advantages that facilitated the repeated diversification of histones. The similarity of *Arabidopsis* and animal H3.3 incorporation patterns is consistent with a general association of H3.3 with several eukaryotic chromatin remodeling processes. The presence of H3.3 on active as well as on many inactive plant promoters of strongly regulated genes suggests a function of H3.3 in transcriptional regulation.

## Materials and methods

### Plant material

All experiments used *Arabidopsis* (*Arabidopsis thaliana*) accession Columbia-0 plants. To produce *35S:H3.3-YFP* lines the cDNA of *HTR4* (*At4g40030*) was fused to the cauliflower mosaic virus (CaMV) 35S promoter at the amino terminus and the YFP cDNA sequence at the carboxyl terminus, and the fusion construct was inserted into the binary vector pCambia1380. Cloning and amplification of the plasmid was done in *Escherichia coli* DH5α. The plasmid was transformed into *Agrobacterium tumefaciens* (strain C58C1) and then transformed into *Arabidopsis* using the floral dip method. Transformants were selected on Murashige and Skoog medium agar plates containing hygromycin. Experimental plants were grown on soil at 21°C in dark (16 h) and 20°C in light (8 h). Plant age was recorded as days after imbibed seeds were sown on soil and transferred to the growth chamber. Leaves (leaf number 6 from about five plants per sample) were harvested after 35 days at *zeitgeber* time 7 (that is, 7 h after start of the photoperiod), and frozen in liquid nitrogen. Note that cell division and expansion had ceased at this developmental stage in the harvested leaves. The experiment was performed with three independent biological replicates.

### RNA expression analysis and protein blots

Expression analysis of the H3.3 transgene was performed as described [22] using gene-specific primers and Universal Probe Libraries (Roche, Basel, Switzerland); Table S2 in Additional file 2) on an ABI Prism 7700 Sequence Detection system (Applied Biosystems, AB, Foster City, CA, USA). The experiment was performed in duplicates. Gene expression levels were normalized to *PP2A*.

For protein immunoblots, 50 mg of frozen *35S:H3.3-YFP* seedlings were ground and the powder was extracted with Buffer M (10 mM Tris-(hydroxymethyl)-aminomethan pH 7.5; 0.5% IGEPAL CA 630; 1% Triton X-100; EDTA free protease inhibitor cocktail (Roche)) plus 150 mM NaCl for 10 minutes at 4°C. The suspension was centrifuged at 16,100 × *g* at 4°C for 10 minutes. The pellet was subsequently extracted using Buffer M containing 500 mM NaCl, centrifuged again, and extracted once more with Buffer M containing 2 M NaCl. Extracted proteins were separated using SDS-PAGE. Total protein was transferred to PVDF-membrane (Carl-Roth, Karlsruhe, Germany). The H3.3-YFP fusion protein was detected using anti-GFP antibody (mouse monoclonal, #11 814 460 001, Roche) and horseradish peroxidase (HRP)-coupled anti-mouse antibody (#115-035-003, Jackson ImmunoResearch Europe Ltd., Newmarket, Suffolk, UK), and was visualized using Immun-Star HRP Substrate (Bio Rad, Berkeley, CA, USA).

### Nuclei preparation, immunostaining and confocal microscopy

Seeds of the *35S:H3.3-YFP* line were germinated and grown for 3 days in Petri dishes on wet filter paper. For visualizing nuclear DNA in live cells, 1 μM of DRAQ5 (eBioscience, Vienna, Austria) was applied to *Arabidopsis* roots for 5 to 10 minutes with vacuum to facilitate penetration. DRAQ5 stain and YFP signals in roots were consecutively analyzed using a Zeiss 710 confocal laser scanning microscope.

For immuno-staining, seedlings were fixed for 20 minutes with ice-cold 4% (w/v) paraformaldehyde in MTSB buffer (50 mM PIPES, 5 mM MgSO_4_, 5 mM EGTA, pH 6.9). Root tips were digested for 10 minutes at 37°C with a PCP enzyme mixture (2.5% pectinase, 2.5% cellulase Onozuka R-10, 2.5% Pectolyase Y-23 (w/v) dissolved in MTSB) and squashed in a drop of MTSB buffer. Immunostaining was performed as described [58]. H3.3-YFP was detected with rabbit anti-GFP (1:100; #A11122, Molecular Probes, Eugene, OR, USA) and donkey anti-rabbit Rhodamine (1:200; #31685, ThermoScientific, Waltham, MA, USA). Pol II was detected using mouse anti-Pol II (1:100; #ab817, Abcam, Cambridge, England) and goat anti-mouse Dylight488 (1:200; #35503, ThermoScientific). For confocal laser scanning microscopy, *35S:H3.3-YFP* seedlings were grown on Murashige and Skoog medium for 5 days before YFP signals in roots were analyzed using a Zeiss 710 confocal laser scanning microscope (Carl Zeiss, Oberkochen, Germany).

### ChIP-qPCR and ChIP-chip

Native ChIP was performed as described [59] with minor modifications. Crude nuclei extracts were produced by treating 100 mg of frozen leaf powder in Nuclei Extraction Buffer (NEB; 20 mM PIPES-KOH pH 7.6, 1 M hexylene glycol, 10 mM MgCl_2_, 0.1 mM EGTA, 15 mM NaCl, 60 mM KCl, 0.5% Triton-X, 5 mM β-mercaptoethanol and EDTA-free protease inhibitor cocktail (Roche)) for 15 minutes at 4°C. The homogenate was filtered through Miracloth (Calbiochem, Nottingham, UK), and a nuclei pellet was collected by centrifugation for 10 minutes at 1,500 × *g* at 4°C. Isolated nuclei were washed once in MNase buffer (50 mM Tris-HCl pH 8, 10 mM NaCl, 5 mM CaCl_2_, and EDTA-free protease inhibitor cocktail (Roche)), treated with 1.3 μl of RNase A, 30 μg/μl (Sigma-Aldrich, St. Louis, MO) and used for Micrococcal Nuclease (New England BioLabs, Ipswich, MA, USA) digestion for 4 minutes (final concentration 0.2 U/μl) in MNase buffer. The reaction was stopped with 10 mM EDTA. After a centrifugation the supernatant was collected as phase 1 chromatin preparation. The pellet was resuspended in buffer S2 (1 mM Tris-HCl pH 8, 0.2 mM EDTA, and EDTA-free protease inhibitor cocktail (Roche)) for 30 minutes. After centrifugation the supernatant was collected as phase 2 chromatin preparation. The two phases of chromatin preparations were combined and the NaCl concentration was adjusted to 50 mM. The majority of the chromatin was of mononucleosome size (data not shown). Histone H1 was depleted by incubating the chromatin preparation with Sephadex C25-CM resin (Pharmacia, Stockholm, Sweden) for 1 h at 4°C [60]. The Triton-X concentration in the mononucleosomal chromatin was adjusted to 0.1% followed by preclearing using non-immune rabbit IgG (see below) and Dynabeads Protein A (Invitrogen, Carlsbad, CA, USA). One tenth of the precleared mononucleosomal chromatin was kept as input control, and one-quarter was used for each immunoprecipitation with 2.5 μg antibody (MAB3422, monoclonal anti-histone antibody, Upstate/Millipore, Billerica, MA, USA; #A11122, polyclonal anti-GFP antibody, can also recognize YFP, Invitrogen; #I5006 non-immune rabbit IgG, reconstituted in H_2_O, Sigma-Aldrich) and collected with Dynabeads Protein A (Invitrogen). After washing, beads were re-suspended in TE buffer (10 mM Tris-HCl, pH 7.5, 1 mM EDTA), and DNA was extracted using phenol-chloroform extraction and ethanol/salt precipitation. Cross-linked ChIP was performed as described [61]. ChIP was performed in biological triplicates.

qPCR was performed using the ChIP-recovered DNA as template using specific primers and probes (Table S1 in Additional file 2). Recovery for H3.3, histone and non-immune IgG was calculated relative to input signals. H3.3 enrichment was calculated using the anti-GFP immunoprecipitation signal normalized to the anti-histone signal.

DNA amplification was performed using the GenomePlex® Single Cell Whole Genome Amplification Kit (Sigma) followed by purification using MinElute PCR Purification kit (QIAGEN, Hilden, Germany).qPCR was performed for six genomic fragments before and after amplification to control for amplification bias (data not shown). Amplified ChIP DNA was fragmented, labeled and hybridized to Affymetrix AGRONOMICS1 *Arabidopsis* tiling arrays as described [62].

### ChIP-chip data analysis

Background correction and normalization were performed as described previously [62]. ChIP-chip data were normalized using MAT [63] implemented in the Aroma.Affymetrix package [64] with the window size parameter set to 100. To detect nucleosomes, data were smoothed using the Savitzky-Golay method [65]. The properties of the Savitzky-Golay filter ensure that the area under each peak, the position of the extrema and the peak widths will not be changed. Numerical derivatives of smoothed ChIP-chip signals were analyzed to identify nucleosomes. Zeros of the first derivative indicate centers of nucleosomes, zeros of the second derivative indicate borders of nucleosomal peaks (Figure S9 in Additional file 1A). After locating the positions of nucleosomal peaks, we estimated peak height and width by least square fitting of each peak to a parabola, as a simplest suitable analytical shape (Figure S9B in Additional file 1). Estimated peak widths had a pronounced maximum at approximately 150 bp, demonstrating that our approach mainly identified signals of nucleosome size (Figure S9C in Additional file 1). The workflow was organized using the Python programming language; all other analysis was performed in R [66]. Deconvolution of the nucleosome H3.3 incorporation scores was done using the MCLUST package [67]. H3.3 enrichment was calculated by normalizing H3.3-YFP ChIP-chip data to histone ChIP-chip data, while H3.3 density was calculated by normalizing H3.3-YFP ChIP-chip data to input data. Visualization of tiling array data was done using the Integrated Genome Browser [68]. H2A.Z data were from [34]. H3K36me2 and H3K27me3 data were from [29]. Pol II data were from [23]. Expression data from leaves were from [22], and expression data from different organs and developmental stages were from [26]. *P*-values were calculated using Wilcoxon’s signed rank test.

### Data availability

Supplementary raw data are available in ArrayExpress [69], accession number E-MTAB-1685. Pol II data [23] are available in the Gene Expression Omnibus [70], accession number GSE21673.

## Abbreviations

bp: base pair; ChIP: chrommatin immunoprecipitation; MNase: micrococcal nuclease; PCR: polymerase chain reaction; Pol II: RNA Polymerase II; qPCR: quantitative PCR; TSS: transcription start site; TTS: transcription termination site; YFP: yellow fluorescence protein.

## Competing interests

The authors declare that they have no competing interests.

## Authors’ contributions

HS, WG, LH designed the research; HS, MN, LB, IM, TW performed research; HS, AS, LH analyzed data; HS, WG, LH wrote the paper. All authors read and approved the final manuscript.

## Supplementary Material

Additional file 1: Figure S1 Generation of H3.3-YFP expressing plants. **Figure S2.** ChIP-qPCR confirmation for H3.3 incorporation. **Figure S3.** H3.3 is present at transcribed genes and has a strong 3′ bias. **Figure S4.** Schematic representations of positional relation between test genes and amplicons for qPCR. **Figure S5.** H3.3 incorporation profiles for genes with differential H3.3 nucleosome association. **Figure S6.** Genes with H3.3 nucleosomes in the promoter are strongly regulated upon environmental stress. **Figure S7.** H3K27me3 profiles of Polycomb group target genes and genes with H3.3 in promoters. **Figure S8.***Arabidopsis* H3.3 does not colocalize with H2A.Z. **Figure S9.** Identification of nucleosomes. Click here for file

Additional file 2: Table S1 List of genes with H3.3 nucleosomes in the promoter, around the TTS or both in promoters and around TTSs and list of all H3.3 nucleosomes. **Table S2.** Primers and Universal Probes (Roche) for qPCR used in this study. Click here for file
