# Supplementary material for: Arabidopsis replacement histone variant H3.3 occupies promoters of regulated genes
Source: Genome Biol. 2014 Mar 21;15(4):R62. doi: 10.1186/gb-2014-15-4-r62 (PMC4054674; doi:10.1186/gb-2014-15-4-r62)
Supplement: Additional file 1: Figure S1 — Generation of H3.3-YFP expressing plants. Figure S2. ChIP-qPCR confirmation for H3.3 incorporation. Figure S3. H3.3 is present at transcribed genes and has a strong 3′ bias. Figure S4. Schematic representations of positional relation between test genes and amplicons for qPCR. Figure S5. H3.3 incorporation profiles for genes with differential H3.3 nucleosome association. Figure S6. Genes with H3.3 nucleosomes in the promoter are strongly regulated upon environmental stress. Figure S7. H3K27me3 profiles of Polycomb group target genes and genes with H3.3 in promoters. Figure S8.Arabidopsis H3.3 does not colocalize with H2A.Z. Figure S9. Identification of nucleosomes. [file gb-2014-15-4-r62-S1.pdf]

Supplemental Figures

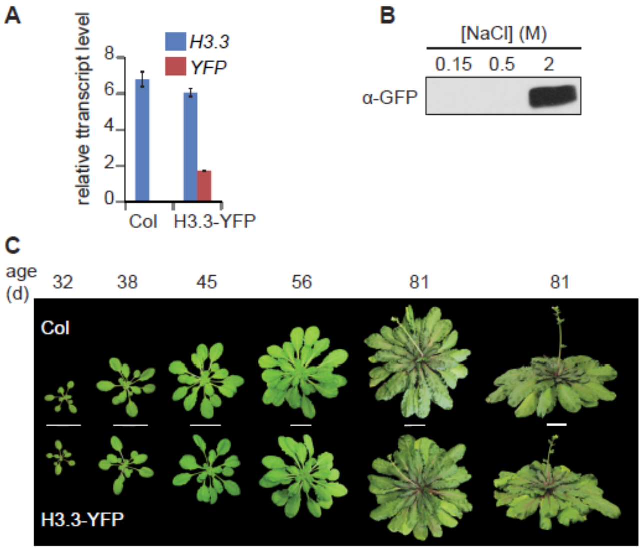

Supplemental Figure S1. Generation of H3.3-YFP expressing plants. (A) H3.3 and transgene expression in wild type (Col) and H3.3-YFP overexpressing lines (H3.3-YFP). **(B)** H3.3-YFP is stably integrated into chromatin. Chromatin was sequentially extracted with increasing salt concentration, and extracted proteins were subjected to SDS-PAGE and immunoblotting with anti-GFP antibodies. **(C)** Development of H3.3-YFP (bottom) and wild-type plants (top) is very similar in SD. Size bars: 5 cm.

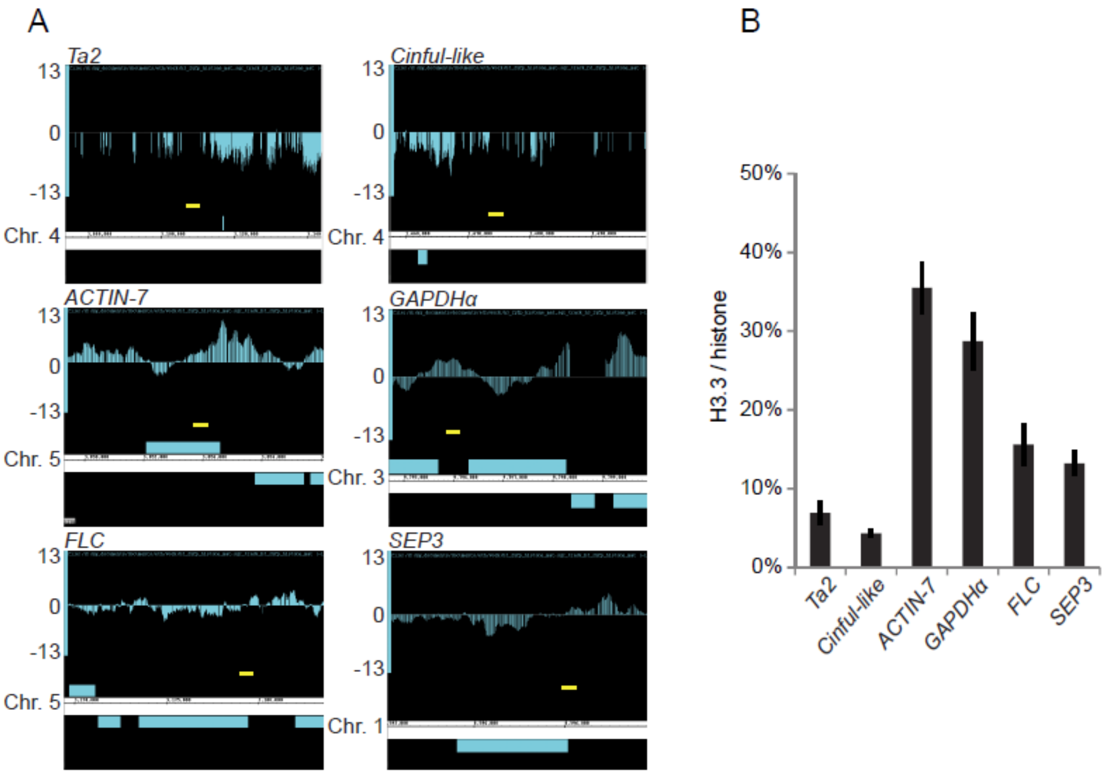

Supplemental Figure S2. ChIP-qPCR confirmation for H3.3 incorporation. (A) Screenshots of ChIP-chip signal profiles around selected loci as visualized in IGB. Yellow short bars indicate approximate locations for the qPCR primer pairs used in (B). **(B)** Q-PCR signals for an independent ChIP experiment detecting H3.3 incorporation.

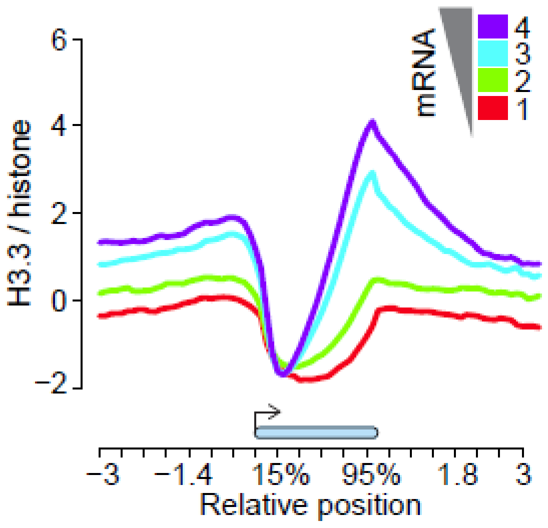

Supplemental Figure S3. H3.3 is present at transcribed genes and has a strong 3' bias. H3.3 incorporation was measured as H3.3-YFP-ChIP normalized to histone-ChIP signals. Metagene plots across gene bodies (blue bar) were constructed between -3 kb and +3 kb. Genes were grouped according to transcript abundance.

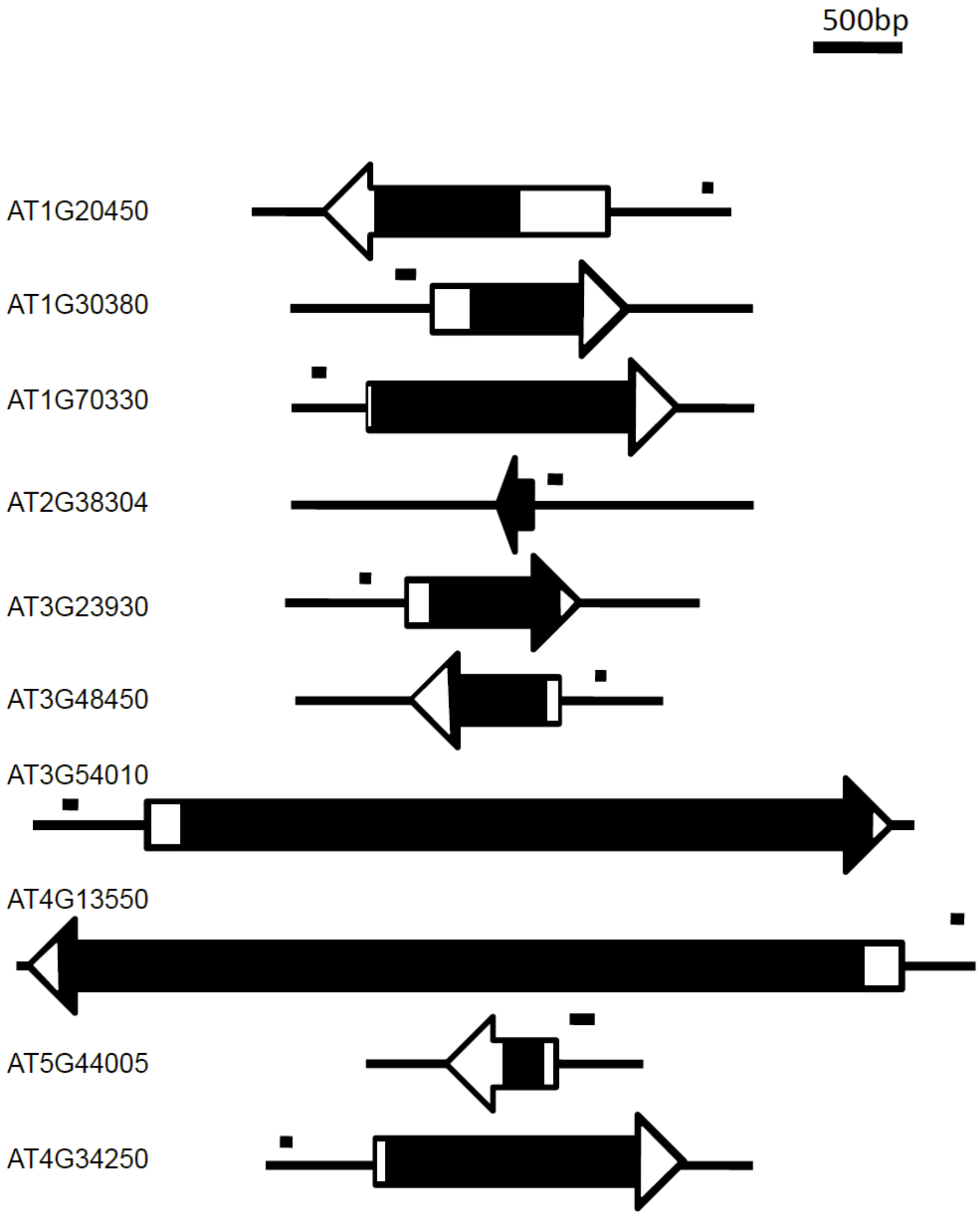

Supplemental Figure S4. Schematic representation of test genes and amplicons for ChIP-qPCR. Arrows represent genes; open boxes represent UTRs. Bold lines show the location of amplicons.

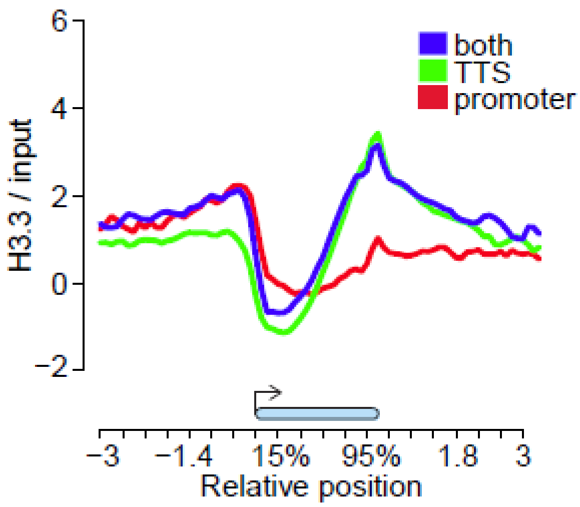

Supplemental Figure S5. H3.3-enrichment profiles after normalisation to input. Metagene plots across gene bodies (blue bar) were constructed between -3 kb and +3 kb for H3.3-signals normalized to input. Genes were grouped according to the presence of H3.3-containg nucleosomes at the promoter (red), close to the TTS (green) or both (blue).

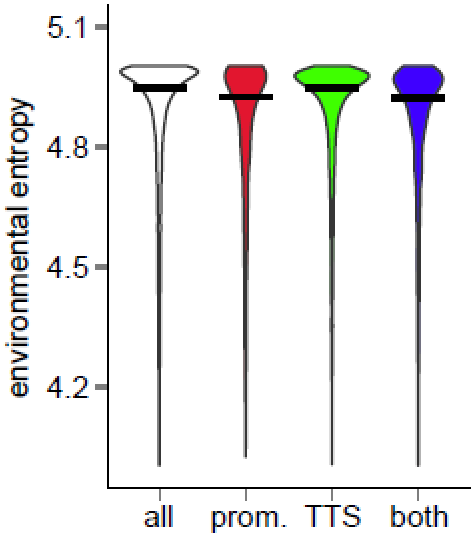

Supplemental Figure S6. Genes with H3.3-enriched nucleosomes in the promoter are strongly regulated upon environmental stress. Expression entropy for all genes (white), genes with H3.3-enriched nucleosomes at the promoter (red), close to the TTS (green) or both (blue). Expression entropy is based on data from (Kilian *et al*, 2007). Horizontal bars indicate median values.

Kilian J, Whitehead D, Horak J, Wanke D, Weinl S, Batistic O, D'Angelo C, Bornberg-Bauer E, Kudla J, Harter K (2007) The AtGenExpress global stress expression data set: protocols, evaluation and model data analysis of UV-B light, drought and cold stress responses. *Plant J* **50**: 347-363

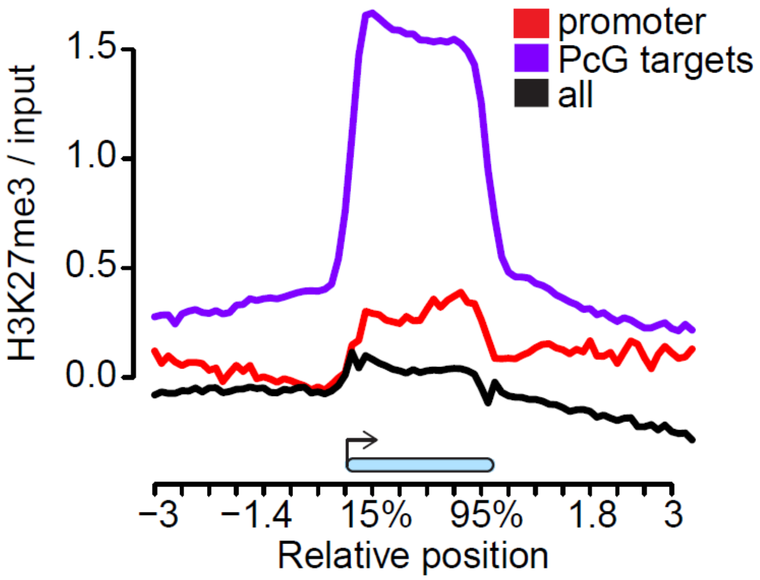

Supplemental Figure S7. Genes with H3.3-enriched nucleosomes in the promoter carry less H3K27me3 than most PcG targets. Metagene plot of H3K27me3 for all repressed genes (black), for repressed genes with promoter H3.3 (red) and for reported PcG target genes (purple) (Oh et al., 2008) across gene bodies (blue bar) between -3 kb and +3 kb. H3K27me3 ChIP-chip data are from (Oh et al., 2008).

Oh S, Park S, van Nocker S (2008) Genic and global functions for Paf1C in chromatin modification and gene expression in Arabidopsis. *PLoS Genet* **4:** e1000077.

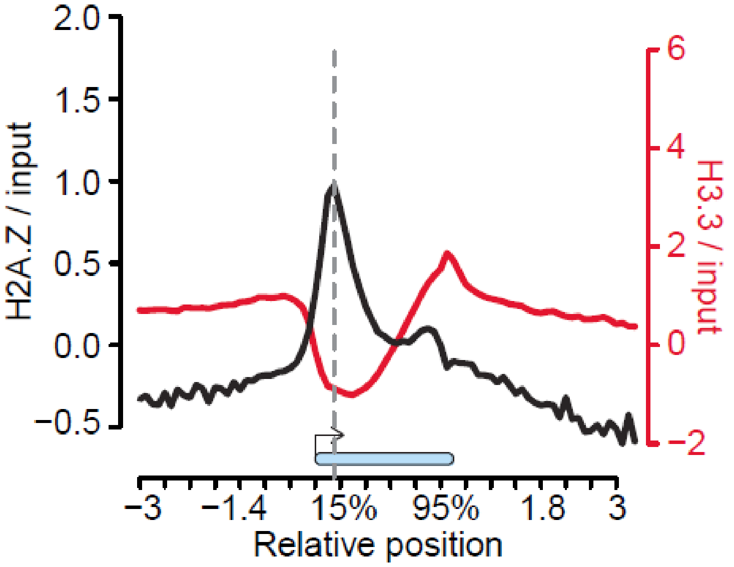

Supplemental Figure S8. Arabidopsis H3.3 does not colocalize with H2A.Z. Metagene plot of H2A.Z (black) and H3.3 (red) across gene bodies (blue bar) between -3 kb and +3 kb for all detectable genes. H2A-Z ChIP-chip data are from Zilberman et al. 2008.

Zilberman D, Coleman-Derr D, Ballinger T, Henikoff S (2008). Histone H2A.Z and DNA methylation are mutually antagonistic chromatin marks. *Nature* **456:** 125-129.

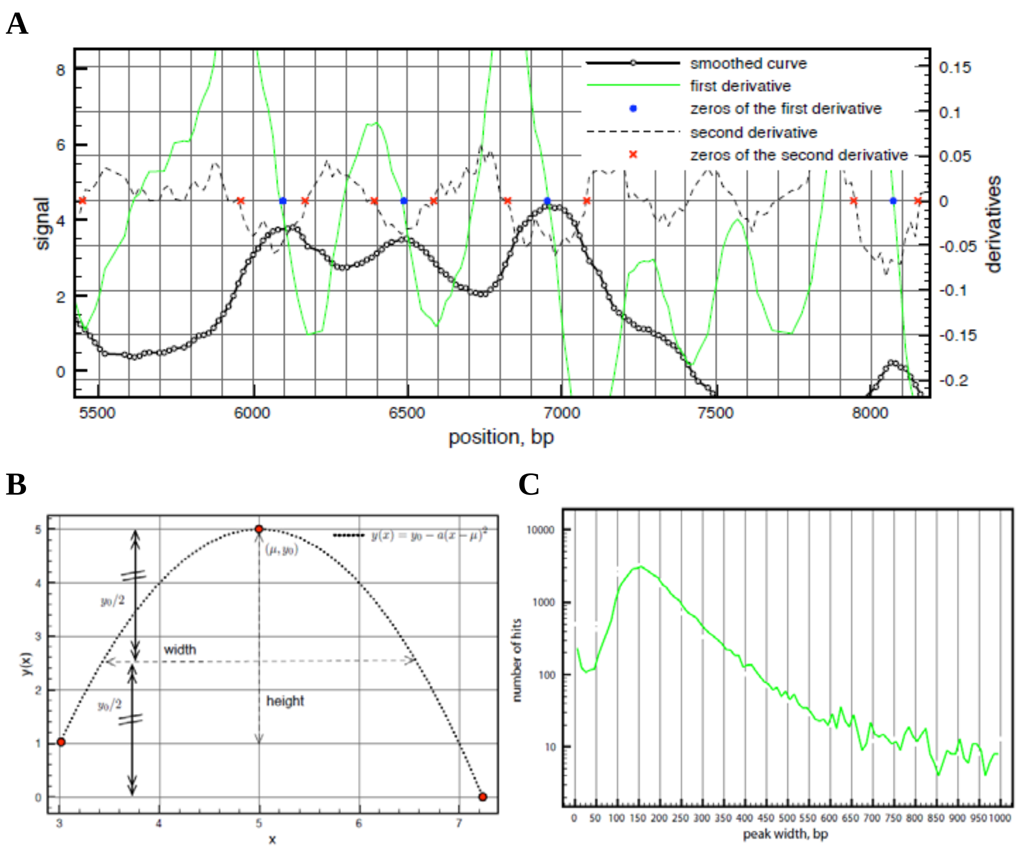

Supplemental Figure S9. Identification of nucleosomes. (A) Nucleosome positions were identified from numerical derivatives of smoothed ChIP-chip signals. The curve with circles is the smoothed signal, the thin line is the first derivative, the thin dashed line is the second derivative. The zeros of the first derivative (blue circles) show positions of the maximum, the zeros of the second derivative (red crosses) show the borders of each peak. **(B)** A parabola $y(x) = y_0 - a(x - \mu)^2$ was fitted to the signals in each identified nucleosome region. A parabola is shown for $y_0 = 5$, $a = 1$, and $\mu = 5$ (dotted curve). The point of maximum (μ, y_0) and bordering points are marked with red-filled circles. The width is estimated as the length of the horizontal dashed arrow at the level $y_0/2$, which is shown as two-headed arrows. The height of the fitted distribution is measured as min[(|$y(\mu)$ – y(left border)|, |$y(\mu)$ – y(right border)|]. **(C)** Distribution of peak widths for chromosome 1. Note the logarithmic y-axis and that most peaks have a width of ~ 150bp.

Supplemental Tables

Supplemental Table S1. Lists of H3.3-containing genes. See separate MS Excel file.

Supplemental Table S2. Primers and Universal Probes (Roche) for q-PCR.

Locus	Primer ID	Primer Sequence	Probe No.
PP2A	LH308	GGAGAGTGACTTGGTTGAGCA	#82
	LH309	CATTCACCAGCTGAAAGTCG	
HTR4	LH1138	CTGCTCGTAAATCTGCACCA	#22
	LH1139	TCTGGTACTTACGGATTTCACG	
YFP	LH1140	GAAGCGCGATCACATGGT	#67
	LH1141	CCATGCCGAGAGTGATCC	
Ta2	LH681	ATGAAAGCGGTCCCATCA	#119
	LH682	CGACTGCTATTCCCTTGTCC	
Cinful-like	LH683	GTCAACGCTGTGGCCAAT	#27
	LH684	TCTTTTGGGTCCTTCGGAAT	
ACTIN-7	LH680	GGAAACATCGTTCTCAGTGGT	#31
	LH782	CTTGATCTTCATGCTGCTAGGT	
GAPDHα	LH676	AATGAAAGGCCCAAAAATCTAA	#134
	LH677	CATGTTTCTATGATCACATATTCACAA	
FLC	LH672	AGCTGACGAGCTTTCTCGAT	#133
	LH673	TTGAGAACAAAAGTAGCCGACA	
SEP3	LH666	ATTGATCTTGTTCTCTATCCTCTTCAA	#103
	LH667	AGAGAGAGAGATTGAGATATCTTTTGG	
AT5G44005	LH1842	CAAAGAATAGTCGAAAGAGCATCA	#157
	LH1843	CACGAGGTAGTTCACTAAAGTGGTAA	
AT4G13550	LH1844	CGAGGAACATCGACGTAGAGA	#146
	LH1845	CAATCTCTGGAAGCAAGTGTTTT	
AT5G01820	LH1846	CGTGGGAGATTTTCTGTTTTG	#143
	LH1847	TCAACTTCTTGTCCACATCTTCA	
AT1G20450	LH1852	GGTAAACACGTGTCAAGAGAACG	#92
	LH1853	AAAACCGGCCATTCAGTTC	
AT3G23930	LH1854	GTCCTTAAACGGTTAAACCTATCTTC	#39
	LH1855	GTGATGGGAGATGTCAGAAGG	
AT3G48450	LH1864	AAGACTAAAAACGTGGATCATGC	#137
	LH1865	AGAAATACTGAATAGGGTAGGTGGAA	
AT2G38304	LH1866	TGGAACTGGATGGTTTGTGA	#147
	LH1867	CAGGCTTAATGGACCCTTTTG	
AT1G30380	LH1868	TCATAGTATTCAAGTTCCATGATTTGT	#165
	LH1869	CATCGTCATCACAGTGCAGAT	
AT4G34250	LH1870	AGTGAACTGATAAACGCCAAAGT	#58
	LH1871	TGGAGACATATACGTATTGAAGTTTTG	
AT1G70330	LH1872	TTATGATAATGCAATGCAACTTGA	#9
	LH1873	TGTCCTACTGTGCTTTTGTCCA	
AT3G54010	LH1876	CATATTTGCTCAACTAACAACTACCAA	#9
	LH1877	TGGATGAACATTTTTGGGTCT	
